# Whole genome sequencing data of *Oryza sativa* L. cv. Takanari

**DOI:** 10.1016/j.dib.2019.104546

**Published:** 2019-09-20

**Authors:** Hiromi Kajiya-Kanegae, Shiori Yabe, Hiroe Yoshida, Kaworu Ebana, Masanori Yamasaki, Hiroshi Nakagawa, Hiroyoshi Iwata

**Affiliations:** a Department of Agricultural and Environmental Biology, Graduate School of Agricultural and Life Science, The University of Tokyo, Japan; b Institute of Crop Science, NARO, Japan; c Institute for Agro-Environmental Sciences, NARO, Japan; d Genetic Resources Center, NARO, Japan; e Food Resources Education and Research Center, Graduate School of Agricultural Science, Kobe University, Japan

**Keywords:** *Oryza sativa* L., Rice, Next-generation sequencing, Long-read sequencing

## Abstract

*Oryza sativa* L. ‘Takanari’ is one of the most productive *indica* cultivars [1,2]. Reciprocal chromosome segment substitution lines (CSSLs) derived from a cross between ‘Koshihikari’ and ‘Takanari’ are useful tools for the detection and precise mapping of target quantitative trait loci (QTL) in ‘Takanari’. Although the available Os-Nipponbare-Reference-IRGSP-1.0 reference genome is available and useful for evaluating genetic diversity among *japonica* cultivars, it is not always useful for evaluating genetic diversity harbored by *indica* cultivars such as ‘Takanari’. To reveal sequence variants in ‘Takanari’ and to exploit these variants in rice breeding programs, the whole genome of ‘Takanari’ was sequenced using a combination of Illumina HiSeq X Ten (20,983,495 reads and %GC 43) and PacBio (2,847,220 high-quality subreads). NGS data obtained have been deposited in the DNA Data Bank of Japan (DDBJ) under accession number DRA007557.

**Table:** Specifications Table

Subject area	*Biology*
More specific subject area	*Agricultural and Biological Sciences, Genomics*
Type of data	*Genome sequence data*
How data was acquired	*PACBIO_SMRT (Sequel) and ILLUMINA HiSeq X Ten*
Data format	*Raw sequencing reads*
Experimental factors	*Whole Genome Sequencing of Oryza sativa L. ‘Takanari’*
Experimental features	*Oryza sativa L. ‘Takanari’ was sequenced with SMRT and short-read sequencing technologies.*
Data source location	*Tsukuba, Japan*
Data accessibility	*The sequence data have been deposited in the DNA Data Bank of Japan (DDBJ) Sequence Read Archive, under submission ID DRA007557.*http://trace.ddbj.nig.ac.jp/DRASearch/submission?acc=DRA007557

**Table:** 

**Value of the data** • Genomic data of high-yielding cultivar 'Takanari' can be useful for identifying genes/quantitative trait loci (QTL) [3]. • The data will help to resolve complex repetitive regions, long indels, and structural variants harbored by *Oryza sativa L.* [5]. • The data is valuable for those working on crop improvement.

## 1 Data

The rice cultivar ‘Takanari’ [1] is a semi-dwarf and high yielding variety developed in Japan [2]. ‘Takanari’ has a large sink size with a high spikelet number per panicle, high source ability and high carbohydrate translocation capacity [6].

Although short read sequencing has high accuracy and throughput, it is difficult to resolve repeat sequences and identify complex structural variations. Additional genomic techniques, such as long-read sequencing [7], are required for detailed characterization of complex genomic regions in rice genomes [4]. This article reports publicly available whole-genome sequences of ‘Takanari’ with single molecule, real-time (SMRT) and short-read sequencing technologies. Using PacBio SMRT technology, we produced 25.6 Gb of long-read sequencing raw data with 68 × coverage. Using Illumina paired-end whole-genome shotgun sequencing technology, we generated 6.3 Gb of short-read sequencing data from a 150 bp paired-end library with coverage of 16 × coverage.

The whole-genome sequences reported in this study will be useful for the genetic improvement of rice, by allowing the genetics of the superior agronomic characteristics of ‘Takanari’ to be investigated.

## 2 Experimental design, materials, and methods

### 2.1 Plant materials

In 2017, *Oryza sativa* L. ‘Takanari’ plants were cultivated in a growth chamber (SANYO MLR-351H) at the National Agriculture and Food Research Organization in Tsukuba, Ibaraki, Japan.

### 2.2 DNA extraction and quality control

Genomic DNA was extracted from the leaf blade of *Oryza sativa* L. ‘Takanari’ using Tiagen Plant Genomic DNA Kit (TIAGEN, China) following the manufacturer's instructions. Quality and quantity of DNA were checked by agarose gel electrophoresis and Qubit Fluorimeter (Thermo Fisher Scientific Inc., USA).

### 2.3 Library preparation and sequencing

The extracted genomic DNA was prepared using SMRTbellTM and TruSeq Nano DNA Library preparation methods. For the PacBio Sequel System, SMRTbell TM libraries were prepared following the manufacturer's protocol, Template Preparation and Sequencing Guide (2010–2014) (Pacific Biosciences of California, CA, USA). For HiSeq X Ten, the TruSeq Nano DNA Library was generated using the Truseq Nano DNA HT Sample Preparation Kit (Illumina USA) following the manufacturer's recommendations.
